# Cardiopulmonary resuscitation knowledge and intention among kindergarten staff in china: a cross-sectional study

**DOI:** 10.3389/fpubh.2025.1696898

**Published:** 2025-12-19

**Authors:** Guangxian Yang, Chao Chen, Min Yang, Xiaorong Xie, Jianghua Fan, Wenwen Fan

**Affiliations:** 1 Hunan Children's Hospital, Changsha, China; 2 Hematology Ward, The Second Xiangya Hospital of Central South University, Changsha, China

**Keywords:** cardiopulmonary resuscitation, kindergarten staff, CPR knowledge, willingness to CPR, training strategy

## Abstract

**Objective:**

Schools are high-risk environments for children's accidents, and teachers, as first responders, play a crucial role in providing timely assistance. Given the low incidence of bystander cardiopulmonary resuscitation (CPR) in China, this study aims to evaluate the CPR knowledge and intention to perform CPR among school staff and identify influencing factors.

**Methods:**

A cross-sectional online survey was conducted from April to June 2022 among 639 kindergarten staff in Changsha, China. The questionnaire evaluated demographics, prior CPR training, knowledge levels, and factors influencing the intention to perform CPR using the Theory of Planned Behavior (TPB).

**Results:**

Among participants, 77.6% reported prior CPR training, predominantly workplace-organized (64.4%) and combining theoretical-practical instruction (72.3%). Knowledge levels averaged 4.7/10, with pronounced deficiencies in AED application (18.8% accuracy). Willingness to perform CPR on strangers was expressed by 71.7% of respondents. Multivariate analysis identified stronger CPR intention among staff aged 35–44 years, those with familial cardiac risk factors, and individuals with superior knowledge (*p* < 0.05). Structural equation modeling revealed that perceived behavioral control (β = 0.371, *p* < 0.001), subjective norms (β = 0.368, *p* < 0.001), and attitudes (β = 0.078, *p* = 0.031) significantly predicted CPR intention (total *R*^2^ = 49.9%), while perceived risk had no significant effect (β = −0.007, *p* = 0.840).

**Conclusions:**

Changsha's kindergarten staff exhibit substantial CPR knowledge, strongly linked to prior training. The findings underscore the necessity for standardized, recurrent CPR education programs and enhanced legal protections to optimize bystander intervention rates in school settings.

## 1 Background

Out-of-hospital cardiac arrest (OHCA) is a significant global cause of mortality. In China, where OHCA incidence is particularly high, over 544,000 people die annually due to this condition. The survival rate for OHCA in China is < 1%, contrasting sharply with the 12% survival rate in the United States. Moreover, the rate of bystander-initiated CPR is notably lower in China (4.5% nationally, with variations such as 11.4% in Beijing and 4.2% in Shanghai) compared to 46.1% in the United States and 29% in Canada, and the quality of these interventions is often suboptimal (1).

Schools, apart from homes, are environments where children are at heightened risk of experiencing OHCA. School personnel frequently become the first responders in such emergencies. Their capacity to deliver immediate and effective aid is pivotal for the child's prognosis. Timely administration of basic life support measures can significantly enhance survival rates; it has been shown that over 80% of children who receive CPR before being transported to the hospital via emergency medical services regain spontaneous circulation (2). There is currently a paucity of data regarding the CPR knowledge and intention to perform CPR among school staff in China. To address this gap, our study focuses on the kindergarten staff in Changsha, a city in central China, to evaluate their CPR knowledge and readiness to perform CPR.

The Theory of Planned Behavior (TPB) proposed by Panchal et al. (3) suggests that the intention to perform CPR by bystanders can be understood through the lens of an individual's attitude toward the behavior, subjective norms, and perceived behavioral control. Behavioral intentions are considered the strongest predictor of actual behavior, influenced by attitudes toward the behavior's outcomes and subjective evaluations of associated risks and benefits. This theoretical framework has been extensively utilized in studies of health-related behaviors and behavioral intentions (4).

This study aims to examine the CPR knowledge, training status, and intention to perform bystander CPR among kindergarten staff in Changsha, China. Additionally, it seeks to identify the factors affecting CPR training uptake and bystander CPR intention using the TPB framework, thereby informing the development of targeted interventions.

## 2 Methods

### 2.1 Design

This study adopted a convenience sampling method. Through collaboration with the local education bureau, online questionnaires were distributed to kindergarten staff in Changsha from April to June 2022. Participants were current kindergarten employees, including teachers, caregivers, administrative staff, and logistics personnel. Prior to completing the questionnaire, all participants were informed of the study's purpose and assured that participation was voluntary, anonymous, and without any repercussions for declining to participate. Access to the collected data was restricted to members of the research team only.

### 2.2 Questionnaires

The research team reviewed relevant literature and convened a panel consisting of three pediatric emergency medicine specialists, three CPR training center experts, and one statistics expert to develop the survey questionnaire (Supplementary File 1). The questionnaire was structured around three main sections:

Demographic Characteristics and Previous Training Experiences: this section covered demographic information such as gender, age, educational level, living arrangements with adult(s) family members, experiences encountering out-of-hospital cardiac arrest (OHCA), and whether CPR had been performed on others. It also included questions about past CPR training experiences, including participation in training, frequency, timing, methods, locations, and duration.

CPR Knowledge: comprised of ten questions, each with only one correct answer. Participants received one point for each correct answer, with a maximum possible score of ten points.

Intention to Perform CPR: based on the Theory of Planned Behavior (TPB), this section developed a scale to measure intention to perform CPR. It included five dimensions: behavioral attitude, subjective norms, perceived behavioral control, perceived risk, and intention to perform CPR. The section consisted of eighteen items, each rated on a five-point Likert scale ranging from 1 (strongly disagree/very unconfident/rarely) to 5 (strongly agree/very confident/most of the time).

### 2.3 Data analysis

Descriptive analysis was performed in *SPSS* 26.0. Structural Equation Modeling (SEM) was performed in *Mplus* Version 7.3. Descriptive statistics were used to summarize the demographic characteristics, previous training experiences, CPR knowledge scores, and intention to perform CPR, along with their influencing factors among the surveyed kindergarten staff. For quantitative variables, means and standard deviations were calculated; for qualitative variables, frequencies and percentages were computed. Chi-square test and *t*-test were used to analyze the differences in demographic and psychological variables between the intervention group and the non-intervention group. SEM was utilized to explore the impact of behavioral attitudes, subjective norms, perceived behavioral control, and perceived risk on the intention to perform CPR. SEM allowed for the examination of both direct and indirect effects within a comprehensive model, providing insights into the complex relationships between these variables and the intention to perform CPR.

## 3 Results

### 3.1 Demographic characteristics of respondents

Among the 639 respondents, there were 35 males (5.5%) and 604 females (94.5%). The average age was 36.28 years (±9.26), with an age range from 17 to 60 years. Educational levels were predominantly at or below the associate degree level, accounting for 63.2% of respondents. Approximately 33.2% of the kindergarten staff lived with adult(s) family members. Only 9.5% of the staff had encountered situations where someone needed CPR, and among these, only 2.2% (14 individuals) had performed CPR on others (Table 1).

**Table 1:** Demographic characteristics of respondents (*n* = 639).

**Variables**	**Characteristics**	**Sample size**	***N* (%)**
Gender	Male	35	5.5
	Female	604	94.5
Age (years)	< 20	15	2.3
	20–34	234	36.7
	35–44	246	38.5
	45–60	144	22.5
Education level	Associate's degree or below	404	63.2
	Bachelor's degree	225	35.2
	Master's degree or above	10	1.57
Job position	Teacher	298	46.6
	Caregiver	211	33.1
	Logistics	69	10.8
	Administration	61	9.5
Work area	Urban	569	89.0
	Rural	70	11.0
Do you live with adult(s) family members?	No	427	66.8
	Yes	212	33.2
Is there a family member with a high risk of coronary heart disease or other conditions that could lead to cardiac arrest?	No	537	84.0
	Yes	102	16.0
Have you ever encountered a situation in your school where a student needed CPR?	No	627	98.1
	Yes	12	1.9
Have you ever encountered a situation in society where someone needed CPR?	No	578	90.5
	Yes	61	9.5
Have you ever performed CPR on someone?	No	625	97.8
	Yes	14	2.2

CPR, cardiopulmonary resuscitation.

### 3.2 Previous training experience

Among the 639 respondents, 477 (74.6%) had previously received CPR training. Of these, 140 individuals (29.4%) had participated in one training session, while only 68 (14.2%) had attended five or more sessions. The timing of their most recent training was distributed as follows: 53.0% within the past year, 27.7% within the past 3 years, and 14.2% within the past 5 years. Regarding the type of training, 27.7% of the staff reported receiving only theoretical training. The most common location for training was at their workplace (64.4%). Additionally, 65.4% of the training sessions lasted < 2 h (Table 2).

**Table 2:** CPR training experience.

**Variables**	**Characteristics**	**Sample size**	***N* (%)**
Previous CPR Training participation (Including online lectures)	Yes	477	74.6
	No	162	25.4
Number of previous CPR trainings	1	140	29.4
	2	127	26.6
	3	96	20.1
	4	27	5.7
	5	19	4.0
	>5	68	14.2
Time of the most recent training	Within the past 1 years	253	53.0
	Within the past 3 years	132	27.7
	Within the past 5 years	92	19.3
Method of the most recent training	Online theoretical training	51	10.7
	Offline theoretical training	81	17.0
	Offline theoretical training + practical training	345	72.3
Location of the most recent training	Hospital	20	4.2
	Community Health Center (Township Health Center)	9	1.8
	Community	18	3.8
	Workplace	307	64.4
	School	112	23.5
	Other	11	2.3
Duration of the most recent training	≤ 1 h	142	29.8
	≤ 2 h	170	35.6
	2–4 h	100	21.0
	>4 h	65	13.6

The results of the univariate analysis, presented in Supplementary Table S1, indicate that female staff, those aged 35–44 years, caregivers, and employees working in urban areas were significantly more likely to have participated in CPR training (*p* < 0.001).

### 3.3 CPR-related knowledge

The average CPR knowledge score among the surveyed kindergarten staff was 4.7 (±2.1) out of a possible 10 points. Notably, 12 individuals (1.9%) scored 0 points, while only 3 (0.5%) achieved a perfect score of 10. Among the specific CPR knowledge items, the question regarding “When to Use an Automated External Defibrillator (AED)” had the lowest correct response rate at 18.8%, whereas the item concerning “CPR Compression Locations for Children and Adults” had the highest correct response rate at 77.3% (Supplementary Table S2).

The univariate analysis revealed that female staff had significantly higher CPR knowledge scores (4.77 points) compared to male staff (3.54 points) (*p* < 0.001). Caregivers scored significantly higher in CPR knowledge (5.36 points) than staff in other positions (*p* < 0.01). Employees who had performed CPR on others had a significantly higher average score (6.21 points) compared to those who had not (4.67 points) (*p* < 0.01). Staff who had never participated in CPR training had an average score of only 3.38 points, significantly lower than those who had participated in training (5.15 points) (*p* < 0.001). These results are detailed in Table 3.

**Table 3:** Univariate analysis of CPR knowledge score.

**Variables**	**Characteristics**	**Knowledge score (*x~ ±s*)**	**t/F**	***p***
Gender	Male	3.54 ± 2.06	−3.407	0.001
	Female	4.77 ± 2.07		
Age (years)	< 20	3.00 ± 1.60	0.792	0.637
	20–34	4.53 ± 1.93		
	35–44	5.02 ± 2.16		
	45–60	4.61 ± 2.12		
Education level	Associate's degree or below	4.69 ± 2.09	0.577	0.833
	Bachelor's degree	4.69 ± 2.04		
	Master's degree or above	5.40 ± 2.91		
Job position	Teacher	4.38 ± 1.91	2.409	0.008
	Caregiver	5.36 ± 1.94		
	Logistics	4.51 ± 2.52		
	Administration	4.20 ± 2.34		
Work area	Urban	4.76 ± 2.10	1.952	0.051
	Rural	4.24 ± 1.90		
Do you live with adult(s) family members?	No	4.68 ± 2.03	−0.377	0.706
	Yes	4.75 ± 2.19		
Is there a family member with a high risk of coronary heart disease or other conditions that could lead to cardiac arrest?	No	4.70 ± 2.08	0.026	0.979
	Yes	4.70 ± 2.14		
Have you ever encountered a situation in your school where a student needed CPR?	No	4.70 ± 2.09	−0.501	0.617
	Yes	5.00 ± 1.81		
Have you ever encountered a situation in society where someone needed CPR?	No	4.72 ± 2.03	0.888	0.375
	Yes	4.48 ± 2.55		
Have you ever performed CPR on someone?	No	4.67 ± 2.06	−2.759	0.006
	Yes	6.21 ± 2.67		
Have you ever attended CPR training, including online and in-person lectures?	No	3.38 ± 2.00	10.063	0.000
	Yes	5.15 ± 1.92		

### 3.4 Factors influencing performing CPR

To identify the key factors influencing bystanders' decision to CPR, this study conducted a between-group comparison of 61 individuals who had encountered a scenario requiring CPR. The sample consisted of 14 individuals in the intervention group (those who performed CPR) and 47 individuals in the non-intervention group. The comparison encompassed demographic variables, CPR knowledge level, and psychological variables based on the Theory of Planned Behavior (TPB) (see Table 4). For continuous variables (e.g., CPR knowledge score, ATT, etc.), independent samples *t*-tests were employed, with the choice of statistic (“Equal variances assumed” or “Equal variances not assumed”) based on the results of Levene's Test for equality of variances. For categorical variables (e.g., gender, work region, etc.), Chi-square tests or Fisher's exact tests were used.

**Table 4:** Comparative analysis of variables between intervention and non-intervention groups (*n* = 61).

**Variables**	***t*/*χ^2^*/Fisher's**	***df***	***p***
Gender	1.215	2	0.544614
Age	0.191	1	0.662343
Work area	0.342	1	0.558558
Job position	2.656	3	0.447755
Education level	12.095	2	**0.002**
CPR knowledge score	−2.876	59	**0.006**
ATT	1.085	10.852	0.301
SN	−0.664	59	0.51
PBC	−0.8	59	0.427
PR	1.448	11.613	0.174
INT	−0.483	59	0.631

Statistical analysis revealed that, among all variables tested, only education level (χ^2^ = 12.095, *df* = 2, *p* = 0.002) and CPR knowledge score [*t*-(59) = −2.876, *p* = 0.006] showed statistically significant differences between the intervention and non-intervention groups. Specifically, the intervention rate was significantly higher in the subgroup with a bachelor's degree compared to the subgroup with an associate degree or below. Furthermore, the CPR knowledge level of participants in the intervention group was significantly higher than that of the non-intervention group. However, no other demographic variables, including gender and age, and none of the psychological variables within the TPB framework—including attitude (ATT), subjective norm (SN), perceived behavioral control (PBC), perceived risk (PR), and behavioral intention (INT)—demonstrated statistically significant differences between the two groups.

### 3.5 Intention to perform CPR

#### 3.5.1 Intention to perform CPR and related influencing factors

The average score for intention to perform CPR among kindergarten staff was 4.18 ± 0.91 (on a 1–5 scale), as detailed in Supplementary Table S3. Univariate analysis results (Table 5) demonstrated that three factors were significantly associated with greater willingness to perform CPR: staff aged 35–44 years (*p* < 0.05), those with family members at high risk of coronary heart disease or cardiac arrest (*p* < 0.05), and participants exhibiting higher CPR knowledge scores (*p* < 0.05).

**Table 5:** Univariate analysis of intention to perform.

**Variables**	**Characteristics**	**Intention to perform (*x~ ±s*)**	**t/F**	***p***
Gender	Male	4.37 ± 0.79	1.25	0.21
	Female	4.17 ± 0.92		
Age (years)	< 20	3.73 ± 1.02	1.64	0.05
	20–34	4.11 ± 0.99		
	35–44	4.28 ± 0.81		
	45–60	4.16 ± 0.92		
Education level	Associate's degree or below	4.16 ± 0.90	0.64	0.87
	Bachelor's degree	4.20 ± 0.95		
	Master's degree or above	4.40 ± 0.79		
Job position	Teacher	4.10 ± 0.97	0.63	0.88
	Caregiver	4.25 ± 0.82		
	Logistics	4.23 ± 0.92		
	Administration	4.23 ± 0.93		
Work area	Urban	4.16 ± 0.92	−1.24	0.22
	Rural	1.31 ± 0.86		
Do you live with adult(s) family members?	No	4.13 ± 0.91	−1.82	0.07
	Yes	4.27 ± 0.92		
Is there a family member with a high risk of coronary heart disease or other conditions that could lead to cardiac arrest?	No	4.13 ± 0.93	−2.78	0.01
	Yes	4.41 ± 0.79		
Have you ever encountered a situation in your school where a student needed CPR?	No	4.17 ± 0.92	−0.78	0.43
	Yes	4.38 ± 0.85		
Have you ever encountered a situation in society where someone needed CPR?	No	4.17 ± 0.89	−0.84	0.40
	Yes	4.27 ± 1.10		
Have you ever performed CPR on someone?	No	4.17 ± 0.91	−1.15	0.25
	Yes	4.46 ± 1.07		
Previous CPR Training Participation (Including Online Lectures)	No	4.20 ± 0.92	0.93	0.36
	Yes	4.12 ± 0.91		
CPR Knowledge Score	Low Score Group (1–5 points)	4.12 ± 0.94	−2.55	0.01
	High Score Group (6–10 points)	4.31 ± 0.82		

Detailed item scores for factors associated with CPR implementation intention are presented in Supplementary Tables S4–S7. Furthermore, Pearson correlation analysis revealed significant positive correlations between CPR implementation intention and behavioral attitudes, subjective norms, perceived behavioral control (all *p* < 0.001), with complete statistical details provided in Supplementary Table S8.

#### 3.5.2 Structural equation model of intention to perform CPR

**Figure 1:**
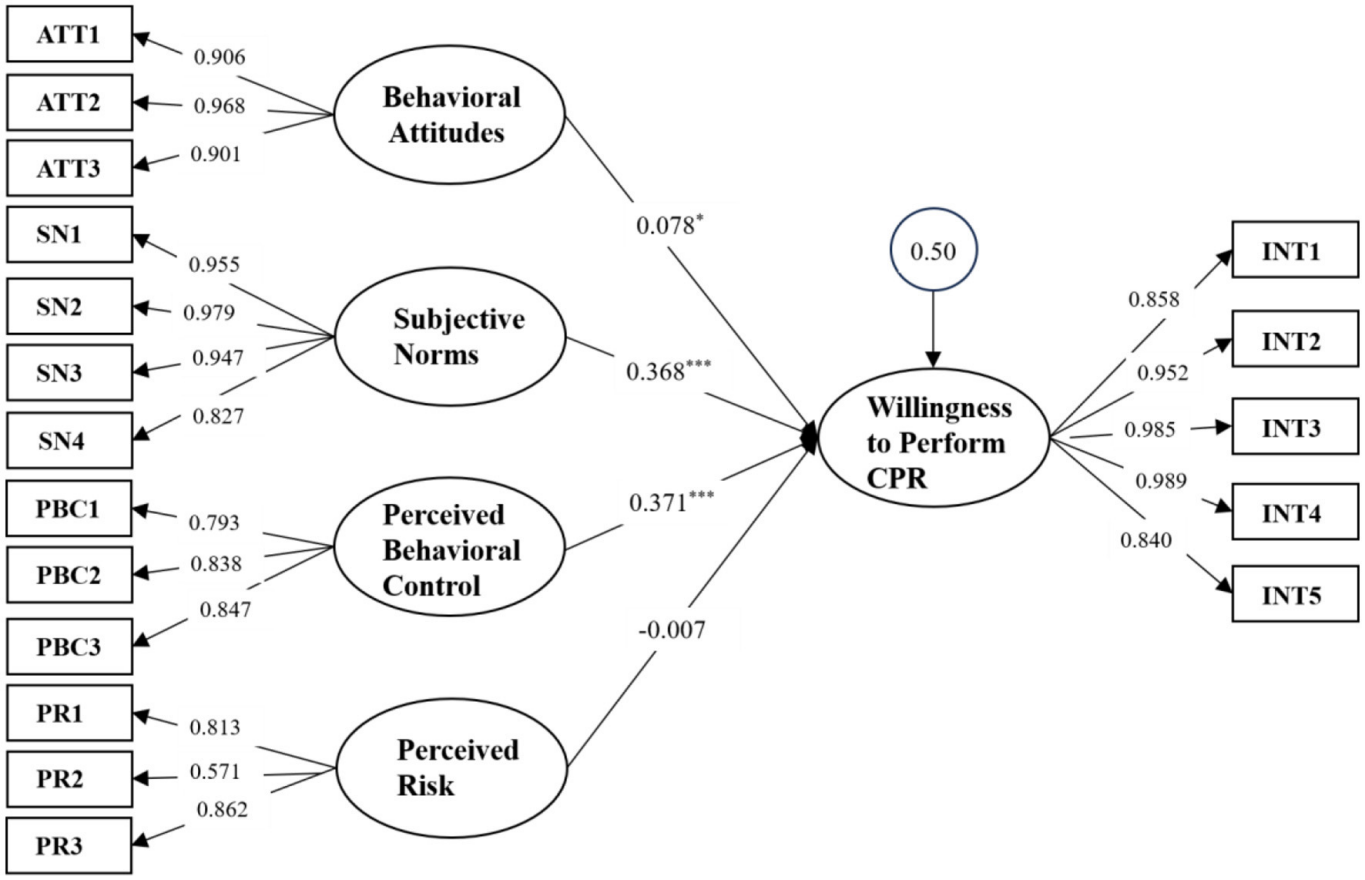
Structural equation model of performation intention.

The latent variables and their corresponding observed variables are detailed in Supplementary Table S9, with the mathematical formulations of the measurement model provided in the Supplementary Table. Intention to perform CPR was designated as the endogenous latent variable, while behavioral attitude, subjective norms, perceived behavioral control, and perceived risk were assigned as exogenous latent variables. The structural hypothesis model is illustrated in Supplementary Figure S1. The analysis confirmed that the model is identifiable, and its evaluation is comprehensively documented in Supplementary Table S10–S12. As demonstrated in Supplementary Table S13 and Figure 1, the findings substantiate three hypotheses (H1, H2, H3). Specifically, behavioral attitude, subjective norms, and perceived behavioral control exerted significant positive influences on intention to perform CPR, with standardized regression coefficients of 0.078, 0.368, and 0.371, respectively. In contrast, perceived risk exhibited a negative, albeit non-significant, effect on intention to perform CPR, with a standardized regression coefficient of −0.007. Collectively, these three latent variables accounted for 49.9% of the variance in kindergarten staff's intention to perform CPR (*R*^2^ = 0.499). Further breakdown revealed that behavioral attitude contributed 1.1%, subjective norms 24.2%, and perceived behavioral control 24.6% to the explained variance (Supplementary Table S14).

## 4 Discussions

This study is the first to systematically investigate the level of cardiopulmonary resuscitation (CPR) knowledge, training status, willingness to perform CPR, and related influencing factors among kindergarten staff in urban China. It provides empirical evidence for understanding the current status and improvement pathways of first-aid capabilities among early childhood educators, and also offers theoretical support for formulating targeted campus first-aid training strategies and policy interventions.

### 4.1 CPR knowledge levels among kindergarten staff in Changsha

The average CPR knowledge score among kindergarten staff in Changsha was 4.7 out of 10 points, surpassing levels reported among civil servants and university students in Chongqing, a city in western China. Notably, 77.6% of respondents had received CPR training—a rate significantly higher than those observed in Chongqing civil servants (21.5%) university students (66.25%) (5), the general public in Hubei Province (24.8%) (6), and the Taiwanese population (64.7%) (7), though lower than Norway's 90% training rate (8). Additionally, 74.6% of participants had undergone training within the past 5 years, exceeding rates in Norway (54%) and international studies (25%−37%) (9, 10). This improvement may be attributed to Hunan Province's “First Responder Initiative,” which promotes on-site emergency care. However, awareness of automated external defibrillator (AED) usage remained critically low (18.8%), reflecting insufficient public education and limited AED deployment in public spaces beyond key locations like subway stations. These findings highlight the urgent need to expand AED accessibility and strengthen practical training in AED application.

### 4.2 Factors influencing CPR implementation

This study identified key determinants of bystander intervention in real-life emergency scenarios by comparing the CPR-performing group (*n* = 47) with the non-intervening group (*n* = 14). The results demonstrated that objective knowledge reserve (CPR knowledge level) and educational background were the core variables predicting intervention behavior, whereas psychological factors from the Theory of Planned Behavior (TPB) and other demographic characteristics showed no significant influence.

Specifically, the CPR knowledge score was significantly higher in the intervention group than in the non-intervention group, indicating that practical skill proficiency serves as the foundation for translating willingness into action (11). In high-pressure environments, good intentions alone are insufficient to trigger behavior; instead, individuals must rely on solid knowledge reserves to overcome uncertainty and hesitation. The significant difference in education level suggests that highly educated groups may be more likely to intervene due to stronger information acquisition abilities, higher self-efficacy, and more rational risk-benefit assessments (e.g., understanding of Good Samaritan laws). Therefore, for populations with lower educational attainment, training strategies should emphasize accessible training materials and clarify legal protections. Notably, none of the TPB psychological variables showed significant differences, challenging the classical hypothesis that “intention directly leads to behavior.” In extremely urgent situations such as cardiac arrest, automated skill responses replace psychological deliberation mechanisms, explaining why high willingness does not necessarily translate into actual action. This study underscores that CPR training should focus on internalizing skills through repetitive practice rather than merely enhancing intention.

### 4.3 Factors influencing intention to perform CPR

#### 4.3.1 Demographic factors

This study further investigates the willingness to perform CPR and its influencing factors. A total of 71.7% of respondents expressed willingness to perform CPR on strangers, with intention levels intermediate between Chinese university students (59.7%) and healthcare professionals (73.9%) (12, 13). Staff aged 35–44 exhibited the strongest intention, potentially linked to occupational stability and family responsibilities, consistent with Dobbie et al.'s (14) findings on age-related behavioral patterns. Participants with family members at high risk of cardiac arrest demonstrated significantly higher willingness, supporting the hypothesis that health risk exposure enhances rescue motivation. Furthermore, those with higher CPR knowledge scores showed a 4.6% increase in intention (4.31 vs. 4.12), validating the “competence-confidence-behavior” theoretical framework.

#### 4.3.2 TPB-based influencing factors

Perceived behavioral control (PBC) emerged as the most influential factor in determining CPR implementation intention, explaining 24.6% of the variance (β = 0.371, *p* < 0.001). This aligns with the Theory of Planned Behavior (TPB), which posits that individuals are more likely to engage in a behavior if they believe they possess the necessary skills and resources (3). However, a critical barrier was identified in low self-efficacy (15, 16), with respondents scoring only 3.46/5 when asked about their confidence in performing CPR without assistance. This lack of confidence may stem from insufficient hands-on practice, as 72.3% of training sessions combined theory with practical components, yet only 14.2% of participants had undergone five or more training sessions. Notably, telephone guidance from emergency medical services (EMS) significantly improved confidence levels (4.01/5, a 15.9% increase). This finding supports Dobbie et al.'s (14) assertion that real-time professional support enhances bystander willingness to act, likely by reducing uncertainty and reinforcing correct technique. These results suggest that future CPR training programs should incorporate simulated emergency scenarios with live feedback, as well as mobile-assisted CPR guidance tools to bridge the gap between training and real-world application.

Subjective norms (SN) were the second strongest predictor (β = 0.368, *p* < 0.001), accounting for 24.2% of the variance in CPR intention. The highest-rated SN item was “societal expectations for first responders to perform CPR on children” (4.18 ± 1.10), indicating that CPR is increasingly viewed as a social responsibility in China. In contrast, family expectations scored lower (4.05 ± 1.13), possibly reflecting lingering concerns about legal or physical risks associated with intervention. This discrepancy suggests that while CPR is broadly endorsed as a public duty, personal networks may still exert cautionary influence. Interestingly, colleague expectations (4.17 ± 1.07) ranked higher than family or friend expectations, implying that workplace culture plays a pivotal role in shaping CPR intentions. These findings highlight the need for institutional policies that normalize CPR as an expected competency among school staff, similar to fire drills or first aid certification.

Despite strong positive attitudes toward CPR (4.53 ± 1.01 overall), this factor contributed minimally to behavioral intention (β = 0.078, *p* = 0.031). Respondents overwhelmingly agreed that “CPR is valuable because it saves lives” (4.59 ± 1.02), yet this belief did not translate into significantly stronger implementation intent. This contrasts with U.S. studies where attitudes were the primary driver of CPR intention among university students (17), suggesting cultural differences in decision-making. One possible explanation is that Chinese respondents may view CPR as a collective responsibility rather than an individual choice, rendering personal attitudes less decisive. Alternatively, the near-universal approval of CPR's value may create a ceiling effect, diminishing its discriminatory power in predicting behavior. Regardless, these results imply that CPR promotion campaigns should move beyond awareness-raising and instead focus on removing practical barriers (e.g., skill retention, legal concerns) to convert positive attitudes into action.

Contrary to prior studies (18), perceived risk had no significant effect on CPR intention (β = −0.007, *p* = 0.840). This shift likely reflects the 2021 Civil Code reform, which legally protects bystanders from liability. Previously, 53.2% of Chinese respondents feared legal consequences (18), but our data suggest this concern has diminished. The lowest-rated risk item was “fear of disease transmission” (2.68 ± 1.30), possibly due to post-pandemic hygiene awareness. However, residual hesitancy persists, as evidenced by the higher score for “concern about legal disputes” (3.71 ± 1.12). To fully capitalize on legal protections, public education should emphasize these safeguards and integrate them into CPR training curricula.

### 4.4 Strengths and limitations

While this study provides valuable insights into CPR knowledge and implementation intentions among kindergarten staff in Changsha, several limitations should be acknowledged. The cross-sectional design restricts our ability to establish causal relationships between training experiences and behavioral outcomes. Additionally, convenience sampling (e.g., the exclusion of kindergartens in rural areas) and the relatively small sample size (*n* = 639) may lead to sample representativeness bias, limiting the generalizability of the research results to other regions or populations. The reliance on self-reported measures rather than observed behaviors may also introduce response bias. Despite these limitations, the study offers important baseline data on CPR training effectiveness in Chinese preschool settings, particularly highlighting the need for improved AED education. Future research should employ longitudinal designs with larger, more diverse samples to better understand the long-term retention of CPR skills and the translation of training into real-world emergency response behaviors.

## 5 Conclusions

This study investigated the status of CPR training, knowledge levels, and factors influencing both the implementation of CPR and the willingness to perform CPR among kindergarten staff in Changsha. The results indicated a relatively high participation rate in CPR training among the staff, with an overall moderate level of CPR knowledge. However, significant deficiencies were observed in critical skills such as the use of automated external defibrillators (AEDs). CPR knowledge scores and education level were identified as the primary factors influencing actual CPR implementation, whereas perceived behavioral control and subjective norms emerged as the most significant factors affecting the intention to perform CPR. To effectively translate intention into action, future efforts should focus on optimizing training programs by emphasizing hands-on practice and proficiency in AED operation. It is essential to establish standardized and periodic CPR skill refresher mechanisms to ensure knowledge and skills remain current. Differentiated training schemes should be designed for populations with lower education levels, utilizing more intuitive teaching methods such as videos and simulators. Additionally, legal awareness campaigns and public advocacy should be strengthened to enhance emergency response awareness.

## Data Availability

The original contributions presented in the study are included in the article/Supplementary material, further inquiries can be directed to the corresponding author.
