# Supplementary material for: Cardiopulmonary resuscitation knowledge and intention among kindergarten staff in china: a cross-sectional study
Source: Front Public Health. 2025 Dec 19;13:1696898. doi: 10.3389/fpubh.2025.1696898 (PMC12757323; doi:10.3389/fpubh.2025.1696898)
Supplement: Supplementary file 1 [file Data_Sheet_1.pdf]

Supplemental Materials for

Cardiopulmonary Resuscitation Knowledge and Intention Among Kindergarten Staff in China: A Cross-Sectional Study

Authors' note:

We developed these materials to provide additional technical information and to keep the main manuscript focused.

Table S1 Univariate analysis of CPR training experience

Variables	Characteristics	Participated in training (%)	χ^2	*p*
Gender	Male	51.4	10.549	**0.001**
	Female	76.0		
Age (years)	<20	33.3	17.041	**0.001**
	20-34	72.2		
	35-44	79.3		
	45-60	75.0		
Education level	Associate's degree or below	73.5	2.265	0.322
	Bachelor's degree	77.3		
	Master's degree or above	60.0		
Job position	Teacher	70.1	31.674	**0.000**
	Caregiver	87.7		
	Logistics	59.4		
	Administration	68.9		
Work area	Urban	77.2	17.223	**0.000**
	Rural	74.6		
Do you live with elderly family members?	No	75.4	0.395	0.530
	Yes	73.1		
Is there a family member with a high risk of coronary heart disease or other conditions that could lead to cardiac arrest?	No	74.7	0.001	0.972
	Yes	74.5		
Have you ever encountered a situation in your school where a student needed CPR?	No	74.3	1.872	0.171
	Yes	91.7		
Have you ever encountered a situation in society where someone needed CPR?	No	74.4	0.205	0.650
	Yes	77.0		

Table S2 Knowledge about cardiopulmonary resuscitation

Item	Correct Number	Correct Rate (%)
How to determine if a person is conscious	439	68.7
How to determine if a person has stopped breathing	180	28.2
How to determine if a person's heart has stopped	398	62.3
Correct steps for performing CPR	165	25.8
Frequency of chest compressions for children	202	31.6
Frequency of chest compressions for adults	372	58.2
Depth of chest compressions for children	441	69.0
Position for chest compressions in children and adults	494	77.3
Ratio of compressions to breaths for single rescuer CPR in children	193	30.2
When to use an AED	120	18.8

The average intention to perform CPR among kindergarten staff was 4.18 ± 0.91 (range: 1 - 5). The highest-scoring items were " Intention to perform CPR on friends and colleagues " (mean: 4.26 ± 0.94 and 4.26 ± 0.93, respectively), while the lowest-scoring item was "Intention to perform CPR on strangers" (mean: 4.02 ± 1.03) (Table S3).

Table S3 Item scores for intention to perform CPR

Item	$\bar{x}$±s
INT1 Intention to perform CPR on cardiac arrest patients.	4.14±0.99
INT2 Intention to perform CPR on children in their kindergarten.	4.21±0.96
INT3 Intention to perform CPR on friends.	4.26±0.94
INT4 Intention to perform CPR on colleagues.	4.26±0.93
INT5 Intention to perform CPR on strangers.	4.02±1.03
Average score	4.18±0.91

The mean score for behavioral attitudes toward CPR was 4.53 ± 1.01 (range: 1 - 5). The highest-rated item was "CPR is valuable because it saves lives" (mean: 4.59 ± 1.02), while the lowest was "Performing CPR to save others enhances my sense of social value" (mean: 4.49 ± 1.11) (Table S4).

Table S4 Item scores for behavioral attitude

Item	$\bar{x}$±s
ATT1 Importance of bystander CPR before medical help arrives.	4.50±1.07
ATT2 CPR is valuable because it saves lives.	4.59±1.02
ATT3 Performing CPR to save others enhances my sense of social value.	4.49±1.11
Average score	4.53±1.01

The average subjective norms score was 4.12 ± 1.04 (range: 1–5). The highest-scoring item reflected "societal expectations for first responders to perform CPR on children" (mean: 4.18 ± 1.10), whereas the lowest was "family expectations to perform CPR on children" (mean: 4.05 ± 1.13) (Table S5).

Table S5 Item scores for subjective norms

Item	$\bar{x}$±s
SN1 Family expectations to perform CPR on children.	4.05±1.13
SN2 Friend expectations to perform CPR on children.	4.07±1.12
SN3 Colleague expectations to perform CPR on children.	4.17±1.07
SN4 Societal expectations for first responders to perform CPR on children.	4.18±1.10
Average score	4.12±1.04

The mean perceived behavioral control score was 3.70 ± 0.93 (range: 1–5). Confidence in performing CPR increased significantly with "telephone guidance from emergency medical services (EMS)" (mean: 4.01 ± 0.98), whereas baseline confidence without support scored lowest (mean: 3.46 ± 1.10) (Table S6).

Table S6 Item scores for perceived behavioral control

Item	$\bar{x}$±s
PBC1 Ability to identify CPR-requiring patients	3.62±1.05
PBC2 Confidence in performing CPR without assistance.	3.46±1.10
PBC3 Confidence in performing CPR with EMS telephone guidance.	4.01±0.98
Average score	3.70±0.93

The average perceived risk score was 3.30 ± 1.02 (range: 1–5). Concerns about "legal disputes arising from performing CPR on children" ranked highest (mean: 3.71 ± 1.12), while fear of "disease transmission during CPR" scored lowest (mean: 2.68 ± 1.30) (Table S7).

Table S7 Item scores for perceived risk

Item	$\bar{x}$±s
PR1 Concern about legal disputes from performing CPR on children.	3.71±1.12

PR2 Fear of disease transmission during CPR.	2.68±1.30
PR3 Concern about negative social perceptions if CPR fails.	3.51±1.25
Average score	3.30±1.02

Table S8 Pearson correlations among main variables

	1	2	3	4	5
AT	1				
SN	0.542***	1			
PB	0.366***	0.563***	1		
PR	0.167***	0.106**	0.138***	1	
INT	0.412***	0.661***	0.604***	0.071	1

Note：*** Correlation is significant at the 0.001 level (2 - tailed).

Measurement Model

The latent variables and their corresponding observed variables are presented in Table S9. The measurement relationships are mathematically expressed as follows:

（1） Behavioral Attitudes (ATT):

$$ATT1 = \lambda_{ATT1} \times ATT + \varepsilon_{ATT1}$$

$$ATT2 = \lambda_{ATT2} \times ATT + \varepsilon_{ATT2}$$

$$ATT3 = \lambda_{ATT3} \times ATT + \varepsilon_{ATT3}$$

（2）Subjective Norms (SN):

$$SN1 = \lambda_{SN1} \times SN + \varepsilon_{SN1}$$

$$SN2 = \lambda_{SN2} \times SN + \varepsilon_{SN2}$$

$$SN3 = \lambda_{SN3} \times SN + \varepsilon_{SN3}$$

$$SN4 = \lambda_{SN4} \times SN + \varepsilon_{SN4}$$

（3）Perceived Behavioral Control (PBC):

$$PBC1 = \lambda_{PBC1} \times PBC + \varepsilon_{PBC1}$$

$$PBC2 = \lambda_{PBC2} \times PBC + \varepsilon_{PBC2}$$

$$PBC3 = \lambda_{PBC3} \times PBC + \varepsilon_{PBC3}$$

（4）Perceived Risk (PR):

$$PR1 = \lambda_{PR1} \times PR + \varepsilon_{PR1}$$

$$PR2 = \lambda_{PR2} \times PR + \varepsilon_{PR2}$$

$$PR3 = \lambda_{PR3} \times PR + \varepsilon_{PR3}$$

（5）Intention to Perform CPR (INT):

$$INT1 = \lambda_{INT1} \times INT + \varepsilon_{INT1}$$

$$INT2 = \lambda_{INT2} \times INT + \varepsilon_{INT2}$$

$$INT3 = \lambda_{INT3} \times INT + \varepsilon_{INT3}$$

$$INT4 = \lambda_{INT4} \times INT + \varepsilon_{INT4}$$

$$INT5 = \lambda_{INT5} \times INT + \varepsilon_{INT5}$$

Table S9 Observed variables for latent constructs of intention to perform CPR

Latent Variable	Code	Observed Variables
Behavioral Attitudes	ATT	ATT1
		ATT2
		ATT3
Subjective Norms	SN	SN1
		SN2
		SN3
		SN4
Perceived Behavioral Control	PBC	PBC1
		PBC2
		PBC3
Perceived Risk	PR	PR1
		PR2
		PR3
Intention to Perform CPR	INT	INT1
		INT2
		INT3
		INT4
		INT5

Structural Equation Model

Consistent with the theoretical framework of this study, the structural model is illustrated in Figure S1. Within this model, intention to perform CPR (INT) is specified as the endogenous latent variable, while behavioral attitudes (ATT), subjective norms (SN), perceived behavioral control (PBC), and perceived risk (PR) are modeled as exogenous latent variables. The structural relationships are formally expressed as: INT=$\gamma_1 \times ATT + \gamma_2 \times SN + \gamma_3 \times PBC + \varsigma INT$.

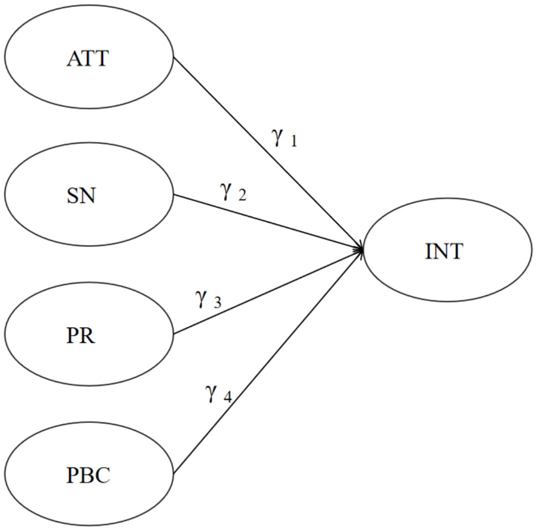

Figure S1 Structural equation model of intention to perform CPR

Model Identification

The structural equation model was statistically identifiable, with $p+q=18$ observed variables, yielding $(p+q)(p+q+1)/2=171$ distinct data points. The model contained 64 freely estimated parameters, satisfying the necessary condition for identification.

Measurement Model Assessment

As shown in Table S10, the composite reliability (CR) values for all latent variables exceeded 0.6, and the average variance extracted (AVE) values exceeded 0.5. Specifically, CR ranged from 0.799 to 0.968, and AVE ranged from 0.577 to 0.863. The factor loadings of the observed variables were all acceptable, ranging from 0.571 to 0.989, with all parameter estimates statistically significant at $p < 0.001$. These results indicate that the latent variables in the model exhibit good internal reliability.

Table S10 Reliability and Convergent Validity

Latent variables	Observed variables	$\bar{x}\pm s$	Factor loadings	p	CR[a]	AVE[b]
Behavioral Attitudes	ATT1	4.50±1.07	0.906	0.000	0.947	0.857
	ATT2	4.59±1.02	0.968	0.000		
	ATT3	4.49±1.11	0.901	0.000		
Subjective Norms	SN1	4.05±1.13	0.955	0.000	0.962	0.863
	SN2	4.07±1.12	0.979	0.000		
	SN3	4.17±1.07	0.947	0.000		
	SN4	4.18±1.10	0.827	0.000		

Perceived Behavioral Control	PBC1	3.62±1.05	0.793	0.000	0.866	0.683
	PBC2	3.46±1.10	0.838	0.000		
	PBC3	4.01±0.98	0.847	0.000		
Perceived Risk	PR1	3.71±1.12	0.813	0.000	0.799	0.577
	PR2	2.68±1.30	0.571	0.000		
	PR3	3.51±1.25	0.862	0.000		
Intention to Perform CPR	INT1	4.14±0.99	0.858	0.000	0.968	0.859
	INT2	4.21±0.96	0.952	0.000		
	INT3	4.26±0.94	0.985	0.000		
	INT4	4.26±0.93	0.989	0.000		
	INT5	4.02±1.03	0.840	0.000		

Note: CR[a]=$(\Sigma\lambda)^2/[(\Sigma\lambda)^2+\Sigma\Theta]$; AVE[b]=$\Sigma\lambda^2/(\Sigma\lambda^2+\Sigma\Theta)$; ΘResidual variances for the observed variables

Additionally, correlation tests among latent variables revealed varying degrees of association, ranging from 0.110 to 0.634. In Table S11, diagonal entries represent the square roots of the average variance extracted (AVE) for each latent variable, while off-diagonal entries indicate correlation coefficients between latent variables. The results demonstrate that all diagonal values exceeded the corresponding off-diagonal correlations, confirming the presence of discriminant validity.

Table S11 Discriminant Validity

Latent variables	Behavioral Attitudes	Subjective Norms	Perceived Behavioral Control	Perceived Risk	Intention to Perform CPR
Behavioral Attitudes	**0.926**				
Subjective Norms	0.534	**0.929**			
Perceived Behavioral Control	0.394	0.613	**0.826**		
Perceived Risk	0.202	0.112	0.157	**0.760**	
Intention to Perform CPR	0.420	0.634	0.624	0.110	**0.927**

Note: Bold values on the diagonal represent the square roots of AVE, while the lower-triangular entries indicate correlation coefficients between latent variables.

Structural Equation Model Assessment

As shown in Table S12, the structural model demonstrated acceptable fit indices: $\chi 2$=599.86; $\chi 2/df$=4.80; TLI=0.955; CFI=0.964; RMSEA=0.077; SRMR=0.040。

Table S12 Model Fit Index

Index	$\chi 2$	χ^2/df	CFI	TLI	RMSEA	SRMR
Criteria*	N/A	1~5	>0.90	>0.90	<0.08	<0.08

Values	599.86	4.80	0.964	0.955	0.077	0.040

Note: *Reference: Bagozzi (1998) and Hu (1999)

Structural Equation Model Interpretation

As shown in Table S13, the results supported three hypotheses (H1, H2, H3). Behavioral attitudes, subjective norms, and perceived behavioral control exhibited significant positive effects on intention to perform CPR, with standardized regression coefficients of 0.078 (p=0.031), 0.368 (p<0.001), and 0.371 (p<0.001), respectively. Perceived risk showed a non-significant negative effect on intention (β=−0.007, p=0.840). Collectively, these three latent variables explained 49.9% of the variance (R^2=0.499) in kindergarten staff's CPR implementation intention. Specifically: behavioral attitudes accounted for 1.1% of the variance, subjective norms contributed 24.2%, perceived behavioral control explained 24.6% (Table S14).

Table S13 Model hypothesis testing

Hypothesis	Path	Coefficient	p	Outcome
H1	Behavioral Attitudes → Intention	0.078	0.031	Supported
H2	Subjective Norms → Intention	0.368	0.000	Supported
H3	Perceived Behavioral Control → Intention	0.371	0.000	Supported
H4	Perceived Risk → Intention	-0.007	0.840	Not Supported

Table S14 Contribution of explanatory variables to variance explanation

Endogenous Variable	Explanatory Variable	Variance Contribution	Total Variance Explained (R^2)
Intention to Perform CPR	Behavioral Attitudes	0.011	0.499
	Subjective Norms	0.242	
	Perceived Behavioral Control	0.246	
	Perceived Risk	0.000	
